# Heritage-specific oral microbiota in Indigenous Australian dental calculus

**DOI:** 10.1093/emph/eoac024

**Published:** 2022-08-05

**Authors:** Matilda Handsley-Davis, Kostas Kapellas, Lisa M Jamieson, Joanne Hedges, Emily Skelly, John Kaidonis, Poppy Anastassiadis, Laura S Weyrich

**Affiliations:** Australian Centre for Ancient DNA (ACAD), School of Biological Sciences, University of Adelaide, Adelaide, SA, Australia; Centre for Australian Biodiversity and Heritage (CABAH), University of Adelaide, Adelaide, SA, Australia; Australian Research Centre for Population Oral Health (ARCPOH), Adelaide Dental School, University of Adelaide, Adelaide, SA, Australia; Australian Research Centre for Population Oral Health (ARCPOH), Adelaide Dental School, University of Adelaide, Adelaide, SA, Australia; Australian Research Centre for Population Oral Health (ARCPOH), Adelaide Dental School, University of Adelaide, Adelaide, SA, Australia; Australian Centre for Ancient DNA (ACAD), School of Biological Sciences, University of Adelaide, Adelaide, SA, Australia; Adelaide Dental School, University of Adelaide, Adelaide, SA, Australia; Adelaide Dental School, University of Adelaide, Adelaide, SA, Australia; Australian Centre for Ancient DNA (ACAD), School of Biological Sciences, University of Adelaide, Adelaide, SA, Australia; Centre for Australian Biodiversity and Heritage (CABAH), University of Adelaide, Adelaide, SA, Australia; Department of Anthropology and Huck Institutes of the Life Sciences, The Pennsylvania State University, University Park, PA, USA

**Keywords:** microbiota, microbiome, Indigenous Australian, Aboriginal Australian, oral health, evolutionary medicine

## Abstract

**Background and objectives:**

Aboriginal Australians and Torres Strait Islanders (hereafter respectfully referred to as Indigenous Australians) experience a high burden of chronic non-communicable diseases (NCDs). Increased NCD risk is linked to oral diseases mediated by the oral microbiota, a microbial community influenced by both vertical transmission and lifestyle factors. As an initial step towards understanding the oral microbiota as a factor in Indigenous health, we present the first investigation of oral microbiota in Indigenous Australian adults.

**Methodology:**

Dental calculus samples from Indigenous Australians with periodontal disease (PD; *n* = 13) and non-Indigenous individuals both with (*n* = 19) and without PD (*n* = 20) were characterized using 16S ribosomal RNA gene amplicon sequencing. Alpha and beta diversity, differentially abundant microbial taxa and taxa unique to different participant groups were analysed using QIIME2.

**Results:**

Samples from Indigenous Australians were more phylogenetically diverse (Kruskal–Wallis *H* = 19.86, *P* = 8.3 × 10^−6^), differed significantly in composition from non-Indigenous samples (PERMANOVA pseudo-*F* = 10.42, *P* = 0.001) and contained a relatively high proportion of unique taxa not previously reported in the human oral microbiota (e.g. Endomicrobia). These patterns were robust to stratification by PD status. Oral microbiota diversity and composition also differed between Indigenous individuals living in different geographic regions.

**Conclusions and implications:**

Indigenous Australians may harbour unique oral microbiota shaped by their long relationships with Country (ancestral homelands). Our findings have implications for understanding the origins of oral and systemic NCDs and for the inclusion of Indigenous peoples in microbiota research, highlighting the microbiota as a novel field of enquiry to improve Indigenous health.

## BACKGROUND AND OBJECTIVES

Around the world, Indigenous peoples experience a disproportionate burden of chronic non-communicable diseases (NCDs), such as diabetes and cardiovascular disease [1–6]. This pattern is prominent in Australia, where Aboriginal Australians and Torres Strait Islanders (hereafter respectfully referred to as Indigenous Australians) experience markedly higher rates of chronic NCDs and lower life expectancy compared to the overall population [4, 7]. Indigenous Australians also disproportionately experience poor oral health [8–11], reinforcing the overall health gap; for example, periodontal disease (PD) increases the risk and severity of diabetes, chronic kidney disease, and cardiovascular disease [12–14].

Complex factors create and sustain these gaps in oral and overall health. A relatively large proportion of Indigenous Australians (39%) live in outer regional and remote areas of Australia, compared to only 9.5% of non-Indigenous Australians [15]. Residents of these areas often experience poor health outcomes, influenced by limited healthcare infrastructure, higher costs of healthcare delivery and a lack of specialist medical practitioners [16]. However, many other factors affect the health of both rural and urban Indigenous Australians, including lack of access to healthcare, mistrust of the Australian government or of healthcare providers, lack of cultural safety in the healthcare system, socioeconomic status and communication barriers between Indigenous patients and healthcare providers [6, 11, 17–21]. Despite increasing knowledge of these causes of the health gap in Australia, government initiatives to address these issues and close the gap have resulted in limited success [6, 7, 22, 23].

Alongside these social, cultural and economic factors, additional mechanisms may also play a role in Indigenous health disparities. Diverse communities of microorganisms (microbiota) live within the human body and play key roles in health and disease, yet they have not been investigated in the context of the Indigenous health gap. In the mouth, oral microbiota are linked with oral diseases, including PD, dental caries and oral cancers [24]. Oral microbiota likely further contribute to the link between PD and increased risk of chronic systemic NCDs [24]. Hence, exploring oral microbiota may reveal new pathways to improve both oral and systemic health outcomes for Indigenous Australians.

However, developing microbiota-based health interventions first requires a detailed understanding of the factors that affect oral microbiota. Oral microbiota are influenced by numerous factors, such as vertical transmission, diet, medical treatments and environmental and behavioural context [25]. However, these factors have only recently been investigated in Indigenous Australians, and only in the context of childhood caries [26, 27]. Furthermore, the impact of the broader evolutionary history of microbiota has not yet been explored in Indigenous Australians. The upheaval of European invasion and rapid industrialization in the past two centuries, following a long period of at least 45 000 years of co-evolution between humans and microbes in Australia, may have perturbed Indigenous Australian microbiota, resulting in an evolutionary mismatch between host and microbiota that contributes to today’s Indigenous health gap [28]. Only a handful of studies to date have investigated the oral microbiota of Indigenous peoples around the world [29–32], while fewer still have explicitly examined the impact of recent and profound lifestyle shifts on the microbiota of Indigenous individuals [28].

We propose that the oral microbiota may be an important, yet critically under-studied, contributor to Indigenous health disparities [24, 28]. We conducted a pilot study to compare oral microbiota preserved in dental calculus (calcified dental plaque)—a long-term record of oral microorganisms in the mouth—from Indigenous and non-Indigenous Australians with PD, as well as from periodontally healthy non-Indigenous individuals. We examine and discuss the possibility for unique microbiota patterns linked to ethnicity or heritage. We use ‘ethnicity’ to express participants’ self-identification as Indigenous Australian or non-Indigenous, as this term encompasses both shared ancestry and culture, and ‘heritage’ to convey the idea of both genetic (human and microbial) and cultural information passed on across generations, without privileging one or the other in our discussion. Furthermore, we explored distinct microorganisms that may be associated with PD in Indigenous Australians, laying the basis for future work identifying microbial mechanisms that may underpin this disease and the broader Indigenous health gap.

## METHODOLOGY

### Ethical approval and consent to participate

Indigenous Australian participants participated in a study on the association between periodontal treatment and cardiovascular health in Indigenous Australians [33]. This study was approved by the Human Research Ethics Committee of the Northern Territory Department of Health and Menzies School of Health Research (09/95), the Central Australian Human Research Ethics Committee (2009.11.05), Northern Territory Correctional Services Research Committee (no number), University of Adelaide Human Research Ethics Committee (179.2009) and the Aboriginal Health Council of South Australia (04-09-311). The collection of samples from non-Indigenous participants was approved by the University of Adelaide Human Research Ethics Committee (H-2012-108). All study participants gave informed consent before participating, and research was conducted in accordance with the World Medical Association Declaration of Helsinki (version VII, 2008).

### Sample collection

Supragingival dental calculus was collected using sterile hand scalers and frozen to await DNA extraction. Samples were collected from Indigenous Australian participants (*n* = 13) living in either Central Australia (CA; *n* = 7) or the Northern Territory’s Top End region (TE; *n* = 6; Table 1). Indigenous Australian participants were aged between 26 and 63 years at the time of sample collection and comprised 5 female and 10 male participants. Samples from periodontally healthy non-Indigenous participants (*n* = 20) were collected at the University of Adelaide Dental Simulation Clinic, and samples from non-Indigenous participants with PD (*n* = 19) at a private dental clinic in metropolitan South Australia (SA; Table 1). Non-Indigenous participants of both sexes were included in the study if they were aged between 20 and 60 years. PD in Indigenous Australian participants was assessed using the 2007 CDC-AAP classification of ‘moderate’ or ‘severe’ periodontitis, as part of inclusion in the aforementioned study investigating periodontal treatment and cardiovascular health [33]. For non-Indigenous participants, guidelines from the 1999 AAP International Workshop for a Classification of Periodontal Diseases and Conditions were used to assess PD.

**Table 1.** Demographic characteristics of study participants

Participant ID	Sampling location	Self-identified ethnicity	PD
A1	TE	Indigenous Australian	Y
A2	TE	Indigenous Australian	Y
A3	TE	Indigenous Australian	Y
A4	TE	Indigenous Australian	Y
A5	TE	Indigenous Australian	Y
A6	TE	Indigenous Australian	Y
A7	CA	Indigenous Australian	Y
A8	CA	Indigenous Australian	Y
A9	CA	Indigenous Australian	Y
A10	CA	Indigenous Australian	Y
A11	CA	Indigenous Australian	Y
A12	CA	Indigenous Australian	Y
A13	CA	Indigenous Australian	Y
1C	SA	Non-Indigenous	N
2C	SA	Non-Indigenous	N
3C	SA	Non-Indigenous	N
4C	SA	Non-Indigenous	N
5C	SA	Non-Indigenous	N
6C	SA	Non-Indigenous	N
7C	SA	Non-Indigenous	N
8Ca	SA	Non-Indigenous	N
9C	SA	Non-Indigenous	N
10C	SA	Non-Indigenous	N
11C	SA	Non-Indigenous	N
12C	SA	Non-Indigenous	N
13C	SA	Non-Indigenous	N
14C	SA	Non-Indigenous	N
15C	SA	Non-Indigenous	N
16C	SA	Non-Indigenous	N
17C	SA	Non-Indigenous	N
19C	SA	Non-Indigenous	N
20C	SA	Non-Indigenous	N
21C	SA	Non-Indigenous	N
19767	SA	Non-Indigenous	Y
19770	SA	Non-Indigenous	Y
19771	SA	Non-Indigenous	Y
19772	SA	Non-Indigenous	Y
19773	SA	Non-Indigenous	Y
19774	SA	Non-Indigenous	Y
19775	SA	Non-Indigenous	Y
19777	SA	Non-Indigenous	Y
19778	SA	Non-Indigenous	Y
19780	SA	Non-Indigenous	Y
19782	SA	Non-Indigenous	Y
19785	SA	Non-Indigenous	Y
19786	SA	Non-Indigenous	Y
19790	SA	Non-Indigenous	Y
19792	SA	Non-Indigenous	Y
19793	SA	Non-Indigenous	Y
19796	SA	Non-Indigenous	Y
19799	SA	Non-Indigenous	Y
19801	SA	Non-Indigenous	Y

Summary of key demographic characteristics of study participants used in oral microbiota analyses: specific sampling location [the Northern Territory’s Top End (TE), Central Australia (CA), or metropolitan South Australia (SA)], self-identified ethnicity (Indigenous Australian or non-Indigenous) and PD status as assessed by an oral health professional [yes (Y) or no (N)].

### DNA extraction, amplification and sequencing

Genomic DNA was extracted from dental calculus in clean laboratory facilities at the Australian Centre for Ancient DNA using an in-house in-solution silica-binding method [34]. Extraction blank controls (EBCs) were processed alongside samples for each extraction, with an average of two EBCs for every five samples. Barcoded amplicon libraries targeting the V4 region of the prokaryotic 16S ribosomal RNA (rRNA) encoding gene region were constructed as previously published [35], with no-template amplification controls processed alongside the biological samples. Double-stranded DNA was quantified for each sample using Qubit (ThermoFisher Scientific). Polymerase chain reaction (PCR) products were pooled at equal relative concentrations, cleaned using Ampure magnetic beads (New England Biolabs) and quantified using TapeStation (Agilent) and quantitative PCR, then combined into a single DNA sequencing library. Paired-end 150 bp sequencing was performed on an Illumina MiSeq at the Australian Genome Research Facility (AGRF).

### Bioinformatic and statistical analysis

Raw BCL data files were converted to FASTQ using bcl2fastq Conversion Software (Illumina), and forward and reverse reads joined with fastq-join (Bioconda). All subsequent data processing and analysis was undertaken using QIIME2 (2020.2 release) [36]. Merged reads were imported into QIIME2, then demultiplexed and quality filtered. Strain-level amplicon sequence variants (ASVs or ‘features’) were obtained using the QIIME2 Deblur plugin. After filtering to remove very low-abundance features (minimum frequency of 10), representative sequences were placed in a 16S rRNA phylogeny (Greengenes 13.8) using the QIIME2 SEPP plugin, and features were taxonomically classified using a pre-fitted Naïve Bayesian classifier trained on the Greengenes 13.8 database.

Alpha diversity (Faith’s phylogenetic diversity [37]) and beta diversity (unweighted UniFrac distance [38]) values were calculated for all dental calculus samples and controls, subsampling at 400 sequences per sample in order to retain a reasonable number of negative controls (*n* = 10). Differences between samples and controls were statistically evaluated using Kruskal–Wallis (for alpha diversity) and permutational multivariate analysis of variance (PERMANOVA) (for beta diversity) tests. Negative controls were subsequently removed from the feature table used for downstream analysis.

Alpha and beta diversity metrics for dental calculus samples only were calculated, visualized and tested for statistical significance as described above, with the subsampling depth increased to 10 000 sequences per sample. Two samples from Indigenous Australians and one sample from a non-Indigenous individual with PD contained fewer than 10 000 sequences and were thus excluded from these diversity analyses. We verified using a lower subsampling depth (1800 sequences per sample) that the two Indigenous Australian samples (A11 and A12) clustered with the other Indigenous Australian samples in Principal Coordinates Analysis (PCoA), consistent with the broad conclusions of this paper (Supplementary Fig. S1). For statistical tests, *P*-values corrected for false discovery rate (FDR) were used for comparisons across more than two groups [39]. After removing features present in <10% of samples, features that differed significantly in abundance across sample groups were identified using the QIIME2 analysis of composition of microbiomes (ANCOM) plugin with default parameters [40]. We identified and characterized features found uniquely in (i) Indigenous Australian samples, (ii) non-Indigenous samples, (iii) samples from the TE and (iv) samples from CA as described in the Supplementary Methods. A detailed record of QIIME2 commands and parameters is provided as Supplementary Material, File S1.

The feature table, sample metadata, taxonomic information, Faith PD values and unweighted UniFrac PCoA results were imported from QIIME2 into RStudio using qiime2R [41, 42]. Figures were constructed using qiime2R, ggplot2 [43] and RColorBrewer [44, 45].

## RESULTS

### Overview of study participants and sample composition

**Figure 1.**
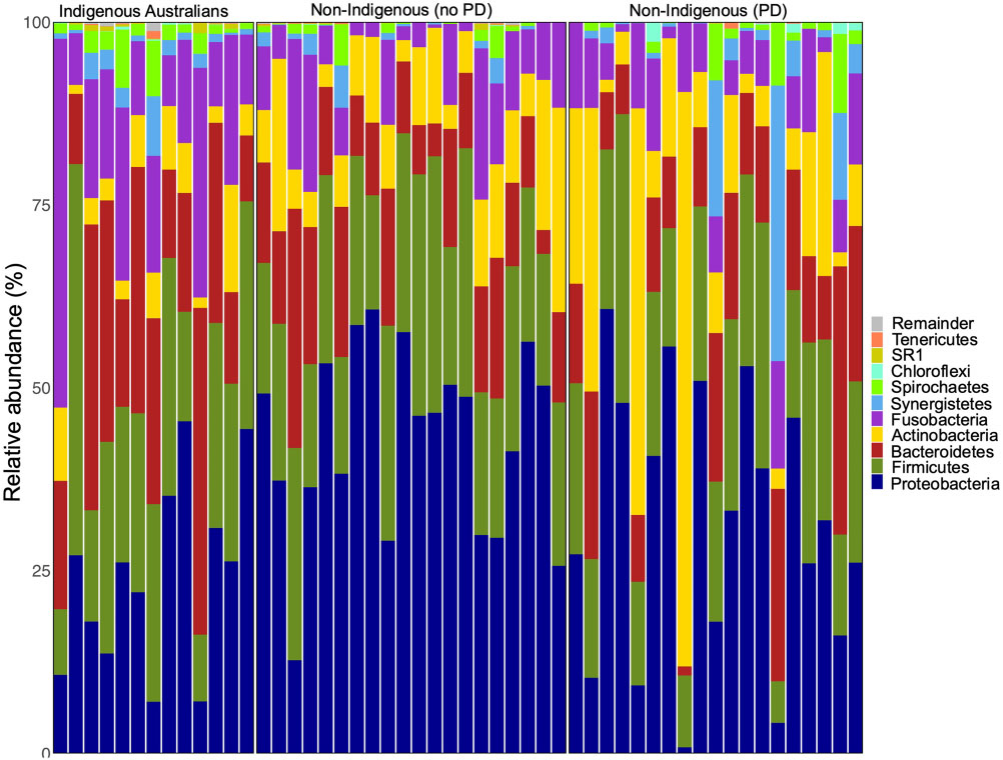
Dental calculus samples are dominated by typical oral taxa. Relative abundance of microbial phyla in all dental calculus samples. Each bar represents a single sample. Samples were dominated by *Proteobacteria*, *Firmicutes*, *Bacteroidetes*, *Fusobacteria* and *Actinobacteria*

Our overall study cohort comprised 52 adult individuals living in Australia (Table 1). The Indigenous Australian group comprised 13 individuals who lived in either CA (*n* = 7) or the TE of the Northern Territory (*n* = 6) and were assessed by an oral health professional as experiencing PD following 2007 AAP-CDC classifications, based on a combination of pocket depth and attachment loss. The non-Indigenous group comprised 39 individuals who lived in SA and did not identify as Indigenous Australian. Of these, 20 individuals were assessed by a dentist as periodontally healthy; the remaining 19 non-Indigenous participants were assessed as experiencing PD using guidelines from the 1999 AAP International Workshop for a Classification of Periodontal Diseases and Conditions, based primarily on clinical attachment loss. In order to avoid discrepancies between metrics across groups, we elected to classify participants into binary ‘periodontal disease’ and ‘periodontally healthy’ categories for our analyses. Overall, dental calculus samples were dominated by typical human oral taxa, such as Proteobacteria (accounting for 35.4% of sequences across all dental calculus samples), Firmicutes (23.9%), Bacteroidetes (16.1%), Fusobacteria (10.9%) and Actinobacteria (9.7%), with 11 remaining phyla contributing ∼4% of total sequences (Fig. 1). We confirmed that samples differed significantly from negative controls (Supplementary Table S1) in both diversity and composition (Supplementary Fig. S2 and Results).

### The diversity and composition of dental calculus microbiota of Indigenous Australian participants differs significantly from that of non-Indigenous participants

**Figure 2.**
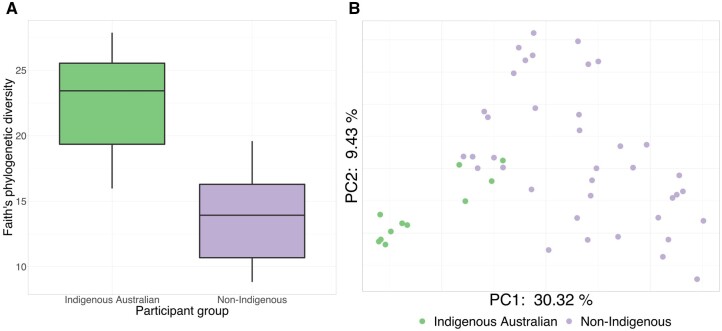
Oral microbiota diversity and composition differ significantly between Indigenous Australian and non-Indigenous individuals. (**A**) Faith’s phylogenetic diversity subsampled to 10 000 sequences per sample. Samples from Indigenous Australians have significantly higher diversity than samples from non-Indigenous individuals (Kruskal–Wallis *H* = 19.86, *P* = 8.3 × 10^−6^). (**B**) PCoA of unweighted UniFrac distances, subsampled to 10 000 sequences per sample. Samples from Indigenous Australians cluster towards one end of PC1 and differ significantly in composition from samples from non-Indigenous individuals (PERMANOVA pseudo-*F* = 10.42, *P* = 0.001)

We initially compared the diversity and composition of all dental calculus samples based on self-reported ethnicity (Indigenous Australian or non-Indigenous). Samples from Indigenous Australians had significantly higher phylogenetic alpha diversity (Kruskal–Wallis *H* = 19.86, *P* = 8.3 × 10^−6^) and differed significantly in composition from the non-Indigenous samples (PERMANOVA pseudo-*F* = 10.42, *P* = 0.001; Fig. 2). To better understand the taxa underlying these differences in diversity and composition, we used ANCOM to identify microbial features (ASVs) that differed significantly in abundance between the Indigenous and non-Indigenous groups. A single feature in the genus *Porphyromonas* was significantly more abundant in Indigenous Australians than in non-Indigenous Australians (*W* = 379). Basic Local Alignment Search Tool (BLAST) searches revealed that the 16S rRNA sequence associated with this feature was a 100% match to multiple *Porphyromonas gingivalis* and unspecified *Porphyromonas* sequences present in the National Centre for Biotechnology Information (NCBI) and Human Oral Microbiome Database (HOMD) databases.

### Indigenous Australians harbour more unique oral microbes than non-Indigenous individuals

We next examined the absolute presence or absence of features unique to either non-Indigenous individuals or Indigenous Australians in this dataset. We identified 125 microbial features that were uniquely found in non-Indigenous individuals and present in at least two samples (Supplementary Table S2); of these, the top five most abundant features were classified as *Streptococcus*, *Rothia aeria*, Peptostreptococcaceae, *Streptococcus anginosus* and *Fusobacterium* taxa. Of the 125 total features, 105 (84%) were known human oral taxa. Of the remaining 20 features, 14 (11% of total) have been identified in studies investigating laboratory contamination and were potentially contaminants [46, 47], leaving six putatively oral but previously unknown unique non-Indigenous features (5% of total). These comprised features in the genera *Blvii28* (*n* = 4), *Dietzia* and *Jeotgalicoccus*. As the non-Indigenous group contained individuals both with and without PD, we also examined the 70 features unique to non-Indigenous individuals with PD only (Supplementary Table S3). The top five most abundant features in this group were classified as *Fusobacteria* (*n* = 2), *Blvii28*, *Neisseria oralis* and *Leptotrichia*. Among the 70 total features, 62 (89%) were known human oral taxa and 5 (7%) were likely contaminants. The three remaining features (4% of total) were all classified as members of the genus *Blvii28*. Overall, the features uniquely found in non-Indigenous individuals were dominated by previously described human oral taxa.

In Indigenous Australians, we identified 171 unique microbial features present in at least two samples (Supplementary Table S4). The top five most abundant features unique to Indigenous Australians were classified as Mogibacteriaceae, *Porphyromonas*, Tissierellaceae, Desulfomicrobiaceae and *Methanobrevibacter*. Altogether, 141 (82%) features were known human oral taxa, while 10 features (6%) were likely contaminants. The remaining 20 features (12%) were putatively oral but previously unknown—more than double the proportion of features in this category uniquely identified in the non-Indigenous group. Of these 20 features, the top five most abundant were classified as *Syntrophomonas*, BS11, ML615J-28 (*n* = 2) and Endomicrobia. Taken together, these results suggest that Indigenous and non-Indigenous individuals may harbour unique strains of oral microbes. The samples from Indigenous Australians contained a relatively high proportion of unique microorganisms not found in current human oral microbiome literature or reference databases.

### Indigenous Australians and non-Indigenous individuals with PD harbour microbiota differences

**Figure 3.**
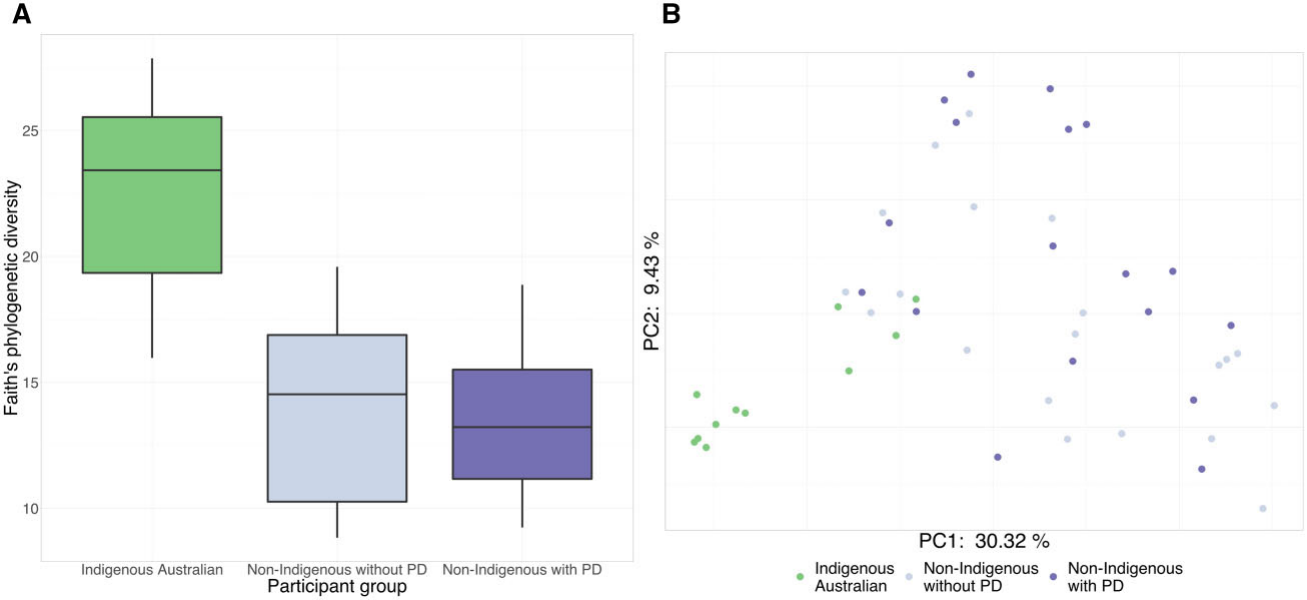
Differences between Indigenous and non-Indigenous oral microbiota are robust to PD status. (**A**) Faith’s phylogenetic diversity subsampled to 10 000 sequences per sample. Samples from Indigenous Australians have significantly higher diversity than samples from both non-Indigenous individuals without PD (Kruskal–Wallis *H* = 15.38, *P* = 1.3 × 10^−4^) and non-Indigenous individuals with PD (*H* = 16.73, *P* = 1.3 × 10^−4^), while diversity of samples from non-Indigenous individuals did not differ significantly according to PD status (*H* = 0.41, *P* = 0.52). (**B**) PCoA of unweighted UniFrac distances, subsampled to 10 000 sequences per sample. Samples from Indigenous Australians cluster towards one end of PC1 and differ significantly in composition from samples from both non-Indigenous individuals without PD (pairwise PERMANOVA pseudo-*F* = 10.14, *P* = 0.0015) and non-Indigenous individuals with PD (pseudo-*F* = 8.96, *P* = 0.0015). A less pronounced, but still significant, difference in composition was observed between non-Indigenous individuals with and without PD (pseudo-*F* = 2.45, *P* = 0.008)

To control for possible biases in our results caused by PD status, we sought to explore the impacts of PD on the oral microbiota differences we observed between Indigenous and non-Indigenous participants. We first divided the samples into three groups according to self-identified ethnicity and PD status: Indigenous Australians with PD (IPD), non-Indigenous individuals with PD (NPD) and non-Indigenous individuals without PD (NH; Fig. 3). Consistent with previous results, samples from Indigenous Australians had significantly higher phylogenetic alpha diversity than samples from non-Indigenous Australians (pairwise Kruskal–Wallis tests: IPD vs. NH H = 15.38, *P* = 1.3 × 10^−4^; IPD vs. NPD *H* = 16.73, *P* = 1.3 × 10^−4^; NPD vs. NH *H* = 0.41, *P* = 0.52; Fig. 3A). Clear and statistically significant clustering according to these categories was observed in PCoA based on unweighted UniFrac distances (pairwise PERMANOVA tests: IPD vs. NH pseudo-*F* = 10.14, *P* = 0.0015; IPD vs. NPD pseudo-*F* = 8.96, *P* = 0.0015; NPD vs. NH pseudo-*F* = 2.45, *P* = 0.008; Fig. 3B). ANCOM testing highlighted the same *Porphyromonas* feature previously identified as significantly more abundant in Indigenous than non-Indigenous samples. This feature was significantly more abundant in IPD than in NPD and was absent from the NH group (*W* = 353).

We next examined the oral microbiota in Indigenous and non-Indigenous individuals with PD, excluding periodontally healthy non-Indigenous individuals. The IPD group had significantly higher alpha diversity than the NPD group (Kruskal–Wallis *H* = 17.1, *P* = 3.5 × 10^−5^; Supplementary Fig. S3A); the two groups again differed significantly in microbiota composition (PERMANOVA pseudo-*F* = 9.26, *P* = 0.001; Supplementary Fig. S3B). Using ANCOM, we identified two features that were significantly more abundant in the IPD group than in the NPD group: the same *Porphyromonas* feature previously identified (*W* = 335) and a Clostridiales feature (*W* = 299). Overall, these results suggest that PD status alone did not drive the microbiota differences observed between Indigenous and non-Indigenous participants. However, this interpretation would be clarified by further research investigating oral microbiota in periodontally healthy Indigenous Australians.

### Oral microbiota diversity and composition may vary across Australian regions

As microbiota differences between Indigenous and non-Indigenous participants were not associated with PD status, we explored other factors that may contribute to unique signatures in the oral microbiota of Indigenous Australians. As heritage is deeply linked to connection to Country (ancestral homelands) for Indigenous Australians, we first investigated geographic differences between our sample collection sites. With only six samples from TE and five samples from CA with sufficient sequencing depth for diversity analyses, we qualitatively assessed differences in oral microbiota of people in different locations and interpret these results with caution. Nevertheless, noticeable differences linked to sampling region were identified across the whole dataset (i.e. differences between the TE, CA or metropolitan SA; Supplementary Fig. S4). Differences in alpha diversity were observed in the oral microbiota of people living in different locations, with CA having the highest diversity and SA (i.e. non-Indigenous individuals) the lowest (Supplementary Fig. S4A). Next, we examined the effect of specific sampling region on microbiota composition, with the same caveats. While the largest differences were between SA and the other two locations (i.e. between non-Indigenous and Indigenous Australian individuals), notable clustering particularly of the samples from CA, while some TE samples cluster with CA and some with SA, can be observed (Supplementary Fig. S4B). Together, these findings suggest that oral microbiota diversity and composition among Australians may be linked to geographic location.

Lastly, we examined microbial features that were uniquely present in samples collected from Indigenous Australians living in a given location (*n* = 6 from TE and *n* = 7 from CA), again with the same caveats due to small sample sizes. Two samples from Indigenous Australian participants living in Central Australia that were excluded from alpha and beta diversity analyses due to lower sequencing depth were retained for this qualitative analysis. We identified only three features unique to the Top End that were found in at least two samples; these were, respectively, classified as members of the order Bacteroidales and the genera *Eikenella* and *TG5* (Supplementary Table S5), which are all known human oral taxa. In contrast, we identified 57 features that were uniquely found in CA and present in at least two samples (Supplementary Table S6). The top five most abundant of these unique features were classified as *Porphyromonas*, Desulfomicrobiaceae, *Leptotrichia*, *Desulfovibrio* and *Peptoniphilus*, with the *Porphyromonas* and Desulfomicrobiaceae features being the same two identified in the top five most abundant unique features in Indigenous Australians overall. Altogether, 49 (86%) of the unique Central Australian features were classified as known human oral taxa and none were classified as likely contaminants, leaving eight features (14%) classified as putatively oral but previously unknown taxa. This last group included features classified as *Syntrophomonas*, BS11, Endomicrobia, OPB56 and a member of the family p-2534-18B5. Several of these were also among the most abundant previously unknown unique features in Indigenous Australians above, implying that many of these features may be localized to the Central Desert. These findings suggest that regional variation may play a role in shaping the oral microbiota among Indigenous Australians. However, we reiterate that due to the small number of samples available from TE and CA, these results with respect to geographic location are preliminary and need further verification.

## CONCLUSIONS AND IMPLICATIONS

This study characterizes the oral microbiota in a cohort of Indigenous Australian and non-Indigenous adults, addressing a critical first step in identifying previously undescribed microbiota-linked mechanisms that may contribute to disparities in Indigenous oral and overall health. Both the diversity and composition of oral microbiota of Indigenous Australians differed significantly from that of non-Indigenous participants, regardless of PD status. Unique microbial features not previously described in the human oral microbiota were also found in Indigenous Australians, and these features differed between Indigenous Australians living in different locations. Together, these results lay the groundwork to better understand how the microbiota influences health and disease.

In our study, oral microbiota of Indigenous Australians had significantly higher alpha diversity and a distinct composition compared to non-Indigenous individuals (Figs 2 and 3), as well as a relatively high proportion of unique taxa not previously observed in the human oral microbiota, including *Syntrophomonas*, BS11, ML615J-28 and Endomicrobia (Supplementary Tables S4–S6). Previous studies of oral microbiota in Indigenous peoples have reported differences in alpha diversity [30–32] and distinct microbiota composition [30, 31, 48] compared to non-Indigenous individuals. However, with the exception of work by Ozga and colleagues [32], these studies typically compare Indigenous and non-Indigenous groups with markedly different lifestyles and subsistence strategies (e.g. industrialized vs. hunter-gatherer), making it difficult to differentiate the impacts of lifestyle (e.g. different diets), environment (e.g. exposure to certain places) and inheritance (e.g. vertical transmission of microbiota adapted to specific lifestyles and environments) independently or concurrently. In contrast, both Indigenous and non-Indigenous participants in our study have industrialized lifestyles. Our findings therefore support the idea that the life and experiences of an individual’s ancestors, and not only current individual lifestyle, may help to shape the oral microbiota [28].

We hypothesize that these differences may be explained by a mechanism of microbiota inheritance across generations, influenced by the evolutionary history of the oral microbiota in different locations. In our study, we found differences in diversity and composition between Indigenous Australian oral microbiota from different sampling locations (the Northern Territory’s TE and CA; Supplementary Fig. S4). Geographic variation in oral microbiota was previously reported among hunter-gatherers and traditional farmers living in the Philippines [30]. Indigenous Australians have close connections to Country: in many cases, the ancestors of living Indigenous Australians lived in a particular location for at least 45 000 years, even if recent colonial disruptions mean that not all Indigenous Australians live on Country today [49]. During this time, Indigenous Australians’ microbiota could have adapted to specific environments or cultural practices such as diets or traditional medicines.

For example, an Endomicrobia feature was uniquely detected in samples from Indigenous Australians in CA (Supplementary Table S6). Endomicrobia species are typically intracellular symbionts that live in the guts of termites and other wood-eating insects [50, 51] and allow the host to digest cellulose. Termites are a traditional food for some Aboriginal Australians in the Central Desert region [52–54], and termite mounds are also used in traditional medicine throughout the Northern Territory [55], suggesting possible mechanisms for the introduction of termite-associated species into the oral microbiota. Therefore, we propose that heritage may play a role in the maintenance of Endomicrobia and other unique oral species in the oral microbiota of Indigenous Australians living industrialized lifestyles. This concept of heritage may encompass factors including transgenerational microbiota inheritance, the experiences of ancestors and ongoing connection to Country and cultural practices, which will require further research to fully understand. Using dental calculus, which preserves microbiota over longer time spans than dental plaque or saliva, may have also provided unique insights into the heritage of oral microbes. We acknowledge that this is only a pilot study with a small number of samples and lacks detailed familial information. Nevertheless, this study opens the door for future research investigating the evolutionary history of unique oral microbes, their connection to heritage, and their roles in health and disease.

While this study was not designed to directly investigate microbial mechanisms that underpin PD, we did observe some possible links between our microbiota data and PD. A microbial feature classified as *Porphyromonas* was significantly higher in abundance in the microbiota of Indigenous Australians. *Porphyromonas gingivalis* is thought to play an important role in tissue destruction during PD progression [56]. This species has been shown to impair innate immunity in mice with knock-on effects for the oral microbiota [57], leading to the hypothesis that *P. gingivalis* is a ‘keystone pathogen’ in PD [58]. Several features uniquely identified in samples from Indigenous Australians (e.g. Mogibacteriaceae, *Porphyromonas*, Tissierellaceae, Desulfomicrobiaceae and *Methanobrevibacter*; Supplementary Table S4) may also be associated with PD [59–64]. This finding may point to the importance of specific strains of periodontal pathogens or unique manifestations of PD in Indigenous Australians, which may be related to a mismatch between the evolution of the oral microbiota in Australia and the lifestyle and environmental exposures experienced by Indigenous Australians today. If so, a deeper understanding of these differences could be important to improve early diagnosis and identify successful treatment options for PD in Indigenous Australians. While outside the scope of this study, the health implications of high diversity and unique composition of oral microbiota in Indigenous Australians are worthy of further investigation.

Building on the results presented here, future work could characterize oral microbiota in more Indigenous Australian communities to better understand how oral microbial communities develop and function in relation to heritage, environment and oral and systemic health. In addition, shotgun metagenomic sequencing could be used to obtain strain-level and functional information, assemble microbial genomes and examine the evolutionary history of the oral microbiota in Australia using ancient dental calculus samples. Furthermore, as existing oral microbiota studies are generally dominated by data from populations in the USA and China [65], updated reference databases that include data from a broader range of people may provide more insights into the global diversity of oral microbiota and its implications for health disparities. As recently suggested by Benezra [66], transdisciplinary research projects in the future should aim to combine scientific and social science approaches to better understand the social, cultural and environmental factors that shape microbiota. Specifically, understanding the impacts of industrialization and shifts in lifestyle on Indigenous oral microbiota and oral health represents a key challenge for future studies. As microbiota research continues to gain clinical relevance, understanding how these microbes function and interact with the host will be crucial to inform the most effective treatment and prevention strategies for NCDs that disproportionately impact Indigenous peoples.

## Supplementary Material

eoac024_Supplementary_Data

## Data Availability

The microbial 16S V4 region amplicon datasets generated using samples from non-Indigenous participants during the current study are available in the Qiita repository (https://qiita.ucsd.edu/) under the Study ID 13416. Study data relating to Indigenous Australian participants are not freely available because of ethical and data protection constraints. These de-identified data are stored at the University of Adelaide and cannot be sent outside the institution. Proposals to access data for further analyses should initially be addressed to Lisa Jamieson (lisa.jamieson@adelaide.edu.au) and will be reviewed by the Indigenous Reference Group and research team.
