# Supplementary material for: Heritage-specific oral microbiota in Indigenous Australian dental calculus
Source: Evol Med Public Health. 2022 Aug 5;10(1):352–62. doi: 10.1093/emph/eoac024 (PMC9400808; doi:10.1093/emph/eoac024)
Supplement: eoac024_Supplementary_Data [file eoac024_supplementary_data.zip › AMP_SupplementaryInfo_final_submission_MHD_20220708.docx]

**Heritage-specific oral microbiota in Indigenous Australian dental calculus**

Matilda Handsley-Davis, Kostas Kapellas, Lisa M. Jamieson, Joanne Hedges, Emily Skelly, John Kaidonis, Poppy Anastassiadis, Laura S. Weyrich

**Supplementary Material**

Supplementary methods

*Sample collection*

Further to information provided in the main text, dental calculus samples collected in Central Australia were initially stored in 1.5mL Eppendorf tubes at -20 ^o^C prior to being transported to Darwin using a liquid nitrogen dry shipper and stored in a -70^o^C freezer. Top End samples were stored in 1.5mL Eppendorf tubes in a -70^o^C freezer immediately following collection. Transport of all Northern Territory samples from Darwin to Adelaide was made with the samples submerged in dry ice and carried on a 3½-hour commercial flight. Samples collected in Adelaide were immediately deposited into a 1.5 mL screw-cap tube and stored in a -20°C freezer to await DNA extraction. A single supragingival calculus sample per individual, taken from either a molar or incisor tooth, was subsequently used for analysis.

*DNA extraction, amplification and sequencing*

Following DNA extraction as described in the main text, the V4 region of the prokaryotic 16S rRNA gene was amplified by polymerase chain reaction (PCR) using the 16S_515F V4 forward primer and barcoded 16S_806R reverse primers (Caporaso et al., 2012). Amplification reactions were set up according to the following specifications: 18.05 µL ultra-pure water, 2.5 µL Platinum Taq HiFi buffer (ThermoFisher), 1 µL MgSO_4_, 0.2 µL dNTPs, 0.25 µL Platinum Taq HiFi DNA polymerase (ThermoFisher), 1 µL each forward and reverse PCR primer, 1 µL genomic DNA extract. PCR was carried out on a BIO-RAD T-100 thermocycler with the following conditions: initial denaturation for 6 minutes at 95°C; 38 cycles of denaturation for 30 seconds at 95°C, annealing for 30 seconds at 50°C, and elongation for 1 minute at 72°C; final elongation for 10 minutes at 60°C. PCR was performed in triplicate for each sample (including EBCs) and pooled following amplification. PCR no-template (blank) controls (NTCs) were processed alongside each amplification, and amplified products were verified by gel electrophoresis. Following quantification, cleaning, and pooling as described in the main text, absolute DNA concentration of each pool was performed by qPCR on a Roche 96 LightCycler using KAPA Illumina qPCR primers, master mix and standards 2, 4, and 6. Sequencing was performed as described in the main text.

*Sequence data processing and feature table construction*

For each sequencing run, raw BCL data files from the sequencing machine were converted to FASTQ using bcl2fastq Conversion Software (Illumina). Forward and reverse reads were joined with fastq-join (Bioconda). All subsequent data processing was undertaken using the QIIME2 command line interface (2020.2 release) (Bolyen et al., 2019). A single multiplexed FASTQ file of merged reads from each sequencing run was imported into QIIME2 as Earth Microbiome Project protocol single-end sequences (EMPSingleEndSequences) using the *qiime tools import* command. For each run, sequences were demultiplexed by sample using the *qiime demux emp-single* command and the unique 16S reverse primer barcode sequences. Sequences were then quality-filtered based on a minimum q-score of 4 using *qiime quality-filter q-score*.

To obtain strain-level amplicon sequence variants (‘features’), sequences from each sequencing run were denoised separately using the *qiime deblur denoise-16S* command with a trim length of 220 base pairs. Resulting feature tables and representative sequences files from each of the three runs were merged into single files for the entire dataset using *qiime feature-table merge* and *qiime feature-table merge-seqs*. Samples that were not part of the current study were removed from the merged feature table using *qiime feature-table filter-samples*. The table was then filtered to remove very low-abundance features using the command *qiime feature-table filter-features* with the *--p-min-frequency* parameter set to 10. Next, the representative sequences file was filtered to retain only sequences from the features that remained in the feature table after filtering, using the command *qiime feature-table filter-seqs* with *--i-table* set as the final merged and filtered feature table. The resulting representative sequences were placed in a 16S phylogeny based on the Greengenes 13.8 database using the *qiime fragment-insertion sepp* command (Janssen et al., 2018). Taxonomy was assigned to the features in the final table using the command *qiime feature-classify classify-sklearn* with a pre-fitted Naïve Bayesian classifier trained on sequences from the Greengenes 13.8 database 99% sequence identity operational taxonomic units (OTUs), trimmed to include only 250 base pairs from the region between the 16S_515F and 16S_806R primers used in this study.

*Bioinformatic and statistical analysis*

All bioinformatic and statistical analysis was carried out using the QIIME2 command line interface (2020.2 release) (Bolyen et al., 2019). Alpha diversity (Faith’s phylogenetic diversity (Faith and Baker, 2007)) and beta diversity (unweighted UniFrac distance (Lozupone and Knight, 2005)) values were calculated for all dental calculus samples and controls using *qiime diversity core-metrics-phylogenetic*, subsampling at 400 sequences per sample in order to retain a reasonable number of negative controls (n=10) for the analysis. The phylogenetic diversity of dental calculus samples and controls was compared using a Kruskal-Wallis test implemented in *qiime diversity alpha-group-significance* (Kruskal and Wallis, 1952). The unweighted UniFrac distances between negative controls and biological samples were evaluated for statistical significance with PERMANOVA tests using *qiime diversity beta-group-significance* (Anderson, 2001). Negative controls were subsequently removed from the feature table used for downstream analysis using the command *qiime feature-table filter-samples*. To further limit the influence of contamination on results, any features identified as interesting or significant in subsequent analyses (i.e. ANCOM or unique features analyses) were cross-referenced against the features found in negative controls to verify that contaminant taxa were not driving the results. Any features identified at higher prevalence in negative controls than in samples were presumed to be laboratory contaminants and excluded from the reported results.

Alpha and beta diversity metrics for dental calculus samples only were calculated, visualised, and tested for statistical significance using the same commands described above. A subsampling depth of 10,000 sequences per samples was selected as a compromise between sufficient sampling depth to calculate accurate diversity metrics and the loss of samples with low sequencing depth. This value led to two Indigenous Australian samples that contained fewer than 10,000 sequences (A11 and A12) being lost from the analysis. We verified using a lower sampling depth (1800 sequences per sample) that these samples clustered with the other Indigenous Australian samples in Principal Coordinates Analysis (PCoA), consistent with the broad conclusions of this manuscript. For statistical tests, an FDR-corrected p-value was used for comparisons across more than two groups (Benjamini and Hochberg, 1995). After filtering the table to remove features present in less than 10% of samples, the command *qiime composition ancom* was used with default parameters to identify features that differed significantly in abundance across sample groups (Mandal et al., 2015).

To investigate the dental calculus microbiota in (a) non-Indigenous samples only and (b) samples from individuals with PD only, the feature table was first filtered using *qiime feature-table filter-samples* with the *--p-where* parameter set to (a) ‘*Culture=”ModernNonIndigenous”’* or (b) *‘Perio=”Y”’* and the above analyses (alpha diversity, beta diversity, and ANCOM differential abundance testing) were repeated as previously described.

To identify features that were found uniquely in either Indigenous Australian or non-Indigenous dental calculus samples, the feature table was filtered to create a table of Indigenous Australian samples and a table of non-Indigenous samples using the command *qiime feature-table filter-samples* with the *--p-where* parameter set to either “*Culture=‘ModernIndigenousAustralian’”* or “*Culture=‘ModernNonIndigenous’”*. Each of the resulting feature tables was then filtered by a list of all the features found in the ‘opposite’ table using *qiime feature-table filter-features*, in order to obtain tables of features that were found either only in Indigenous Australians or only in non-Indigenous Australians. The same process was followed, using the table of feature unique to Indigenous Australians as input, to generate lists of features unique to the Top End and Central Australia. Unique features in each group were further filtered and classified as follows. Following removal of features present in only one sample from the tables using the command *qiime feature-table filter-features* with the *–p-min-samples* parameter set to 2, each table of unique features was exported as a feature-by-sample matrix along with taxonomic classifications (i.e. as a ‘classic OTU table’ format with taxonomy). To better characterise the unique features present in each table, taxonomic classifications (i.e. taxa names) were queried against the expanded Human Oral Microbiome Database (eHOMD) (v15.2 16S rRNA RefSeq) website (Escapa et al., 2018) and thereby classified into the following categories: “in HOMD” (present in eHOMD and at least 50% of hits classified as oral), “unassigned body site” (present in eHOMD but less than 50% of hits classified as oral), or “not in HOMD” (no hits in eHOMD). Taxa classified as “unassigned body site” or “not in HOMD” were then cross-referenced against both the negative controls from this study and two published lists of common laboratory contaminants previously identified in microbiota studies (Eisenhofer et al., 2019; Weyrich et al., 2019). Any features found in either the negative controls or published contaminant lists were classified as likely contaminants (red cells in tables). The remaining taxa not classified as primarily oral in eHOMD or as likely contaminants were then subjected to literature searches using the search terms “[taxa name] oral microbiome” and “[taxa name] human microbiome”. Taxa previously identified in human oral samples in at least two studies were then classified as known oral taxa despite absence from eHOMD (yellow cells in tables), with remaining taxa classified as previously unknown putatively oral taxa (orange cells in tables).

A detailed record of commands and parameters used for data analysis in QIIME2 is provided in File S1.

**File S1. Electronic notebook of QIIME2 commands.** Record of QIIME2 commands used to generate results for ‘Heritage-specific oral microbiota in Indigenous Australian dental calculus’ (MHD_FinalAnalysis_SuppMaterial_FullScripts_final_20201018.txt).

Supplementary results

**
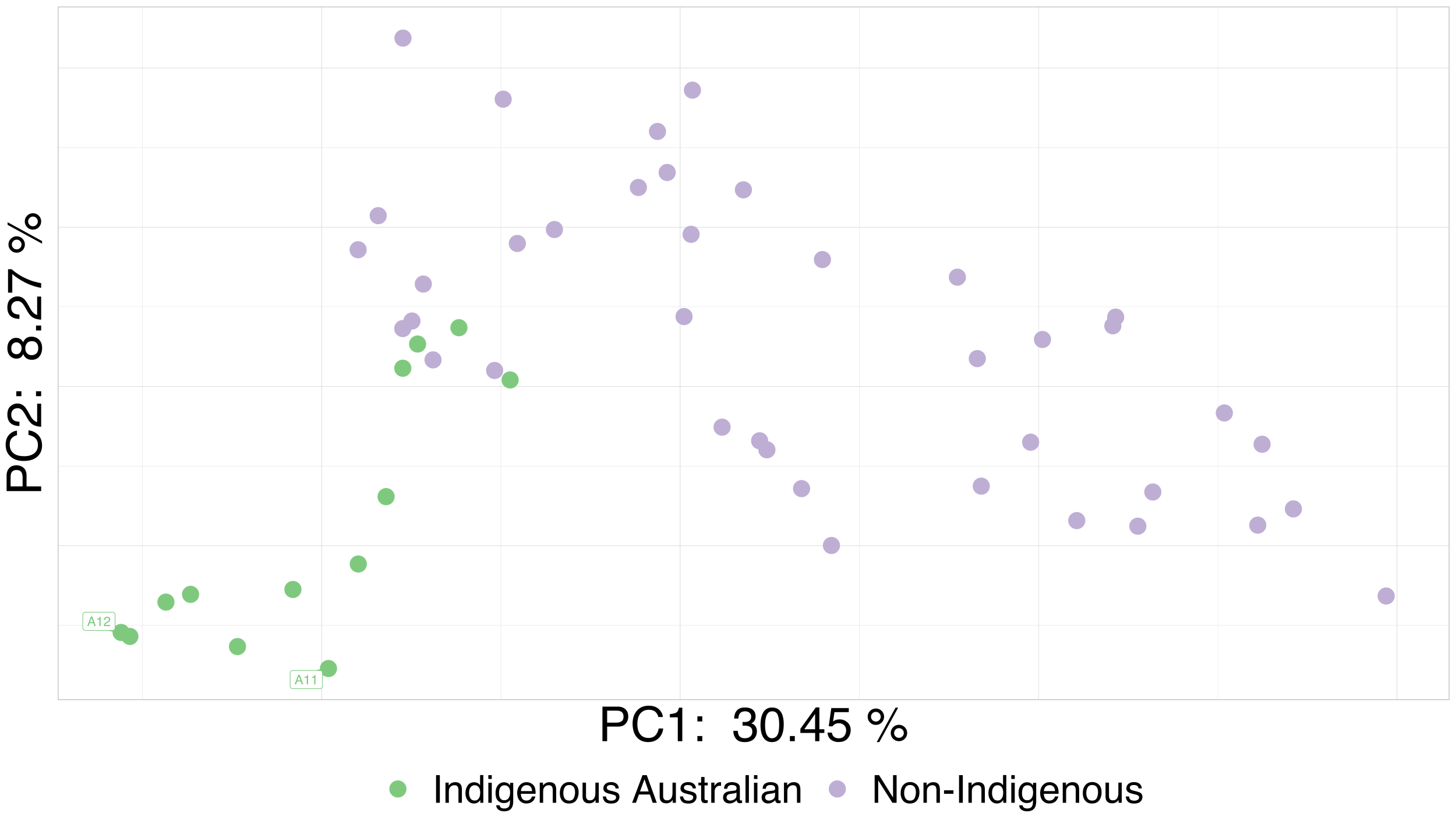
**

**Figure S1. Samples removed due to low sequencing depth cluster with other Indigenous Australian samples.** Principal coordinates analysis (PCoA) of unweighted UniFrac distances, subsampled to 1800 sequences per sample. The samples A11 and A12 were excluded from alpha and beta diversity analyses presented in the main text, as they contained fewer than 10,000 sequences per sample. Nevertheless, these two samples cluster together with other Indigenous Australian samples in PCoA, consistent with the broad findings of the manuscript.

**Typical oral microbiota signal obtained from modern dental calculus samples**

As contamination can substantially impact microbiota data (Eisenhofer et al., 2019; Salter et al., 2014), we tracked background microbial DNA in reagents and the laboratory environment using two types of negative controls: extraction blank controls (EBCs; empty tubes taken through DNA extraction protocol) and amplification no-template controls (NTCs; empty tubes taken through 16S amplification protocol) (Table S1).

**Table S1. Negative controls used for tracking background microbial DNA**

| **Control type** | **Description** | **Purpose** |
| --- | --- | --- |
| Extraction blank control (EBC) | An empty tube taken through DNA extraction protocol alongside dental calculus samples | Track background and contaminant DNA present during DNA extraction |
| No-template control (NTC) | An empty tube taken through 16S PCR amplification alongside dental calculus samples | Track background and contaminant DNA present during PCR amplification of microbial 16S ribosomal RNA gene |

We confirmed that negative controls differed from dental calculus samples in their microbial composition (Figure S2A) and had significantly lower microbial alpha diversity than dental calculus samples (Kruskal-Wallis H=24.76, p=6.5 x 10^-7^) (Figure S2B). Further, the negative controls clustered separately from biological samples in Principal Coordinates Analysis (PCoA) (Figure S2C) and differed significantly in their microbial composition (PERMANOVA pseudo-F=19.09, p=0.001). Taxa commonly found in human oral microbiota were identified across all dental calculus samples. Examples of such widespread oral taxa include the phyla *Proteobacteria* (accounting for 35.4% of sequences across all dental calculus samples), *Firmicutes* (23.9%), *Bacteroidetes* (16.1%), *Fusobacteria* (10.9%), and *Actinobacteria* (9.7%), with 11 remaining phyla contributing approximately 4% of total sequences (Figure 1A). By contrast, the controls were dominated by *Firmicutes* (52.1%), *Proteobacteria* (38.7%), and *Actinobacteria* (7.9%), with 4 remaining phyla contributing approximately 1% of the sequences (Figure S2A). We concluded that we had reconstructed a trustworthy oral microbial signal from modern supragingival dental calculus samples in our dataset.


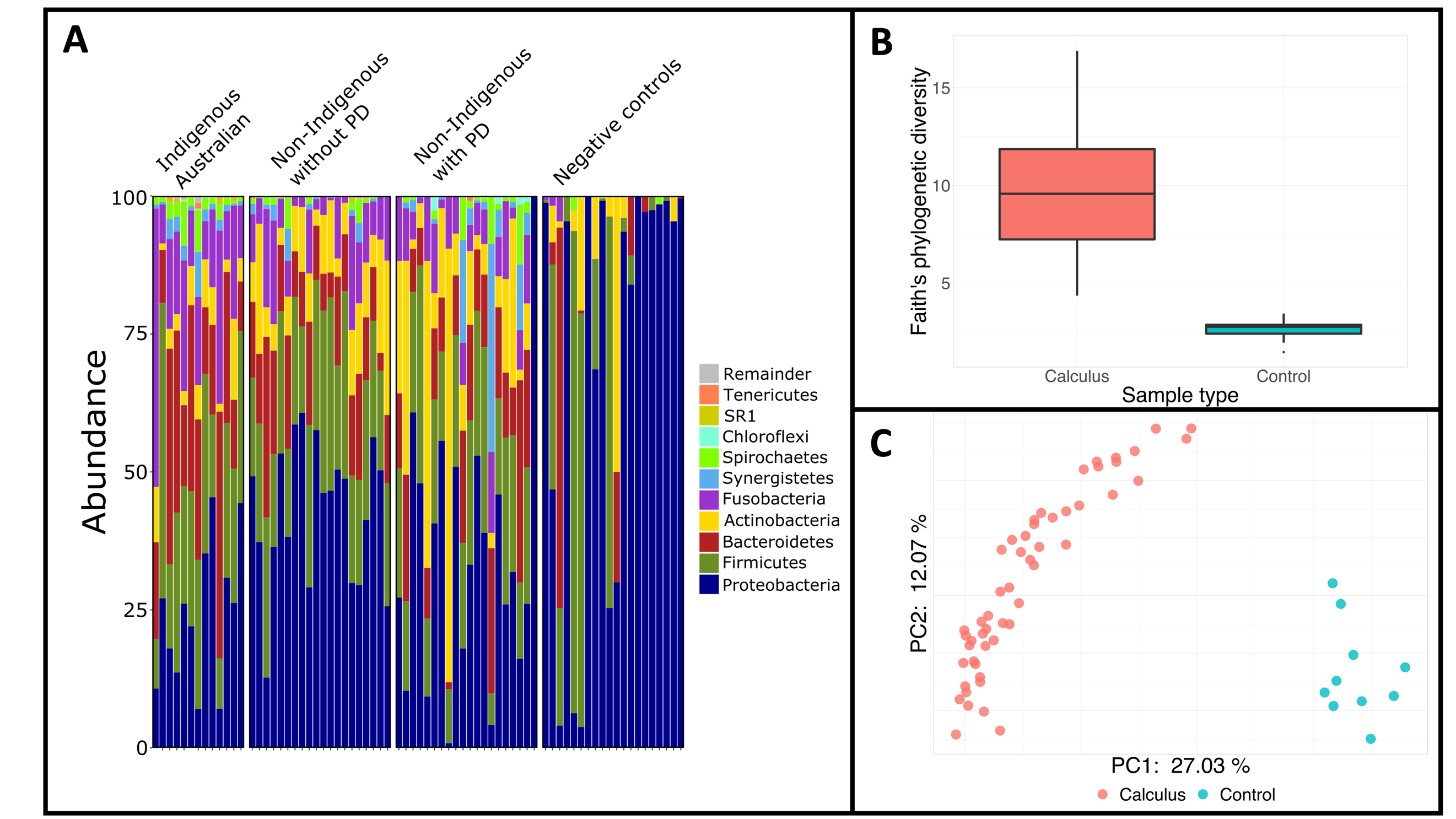


**Figure S2. Microbial diversity and composition differs between dental calculus samples and negative controls.** (A) Relative abundance of microbial phyla in dental calculus and negative control samples. Each bar represents a single sample. Dental calculus samples were dominated by Proteobacteria, Firmicutes, Bacteroidetes, Fusobacteria and Actinobacteria, while negative controls were dominated by Firmicutes, Proteobacteria and Actinobacteria. (B) Faith’s phylogenetic diversity calculated for all dental calculus samples and negative controls, subsampled to 400 sequences per sample. Dental calculus samples have significantly higher alpha diversity (Kruskal-Wallis H=24.76, p=6.5 x 10^-7^). (C) Principal coordinates analysis (PCoA) of unweighted UniFrac distances between all dental calculus samples and negative controls, subsampled to 400 sequences per sample. Dental calculus samples (red) and controls (blue) cluster separately and significantly differ in microbial composition (PERMANOVA pseudo-F=19.09, p=0.001).

**Table S2. List of features uniquely found in non-Indigenous individuals**

Refer to Table S2 Excel spreadsheet (TableS2_NonIndigenous_Unique.xlsx).

**Table S3. List of features uniquely found in non-Indigenous individuals with periodontal disease**

Refer to Table S3 Excel spreadsheet (TableS3_NonIndigenous_Perio_Unique.xlsx).

**Table S4. List of features uniquely found in Indigenous Australians**

Refer to Table S4 Excel spreadsheet (TableS4_IndigenousAustralian_Unique.xlsx).


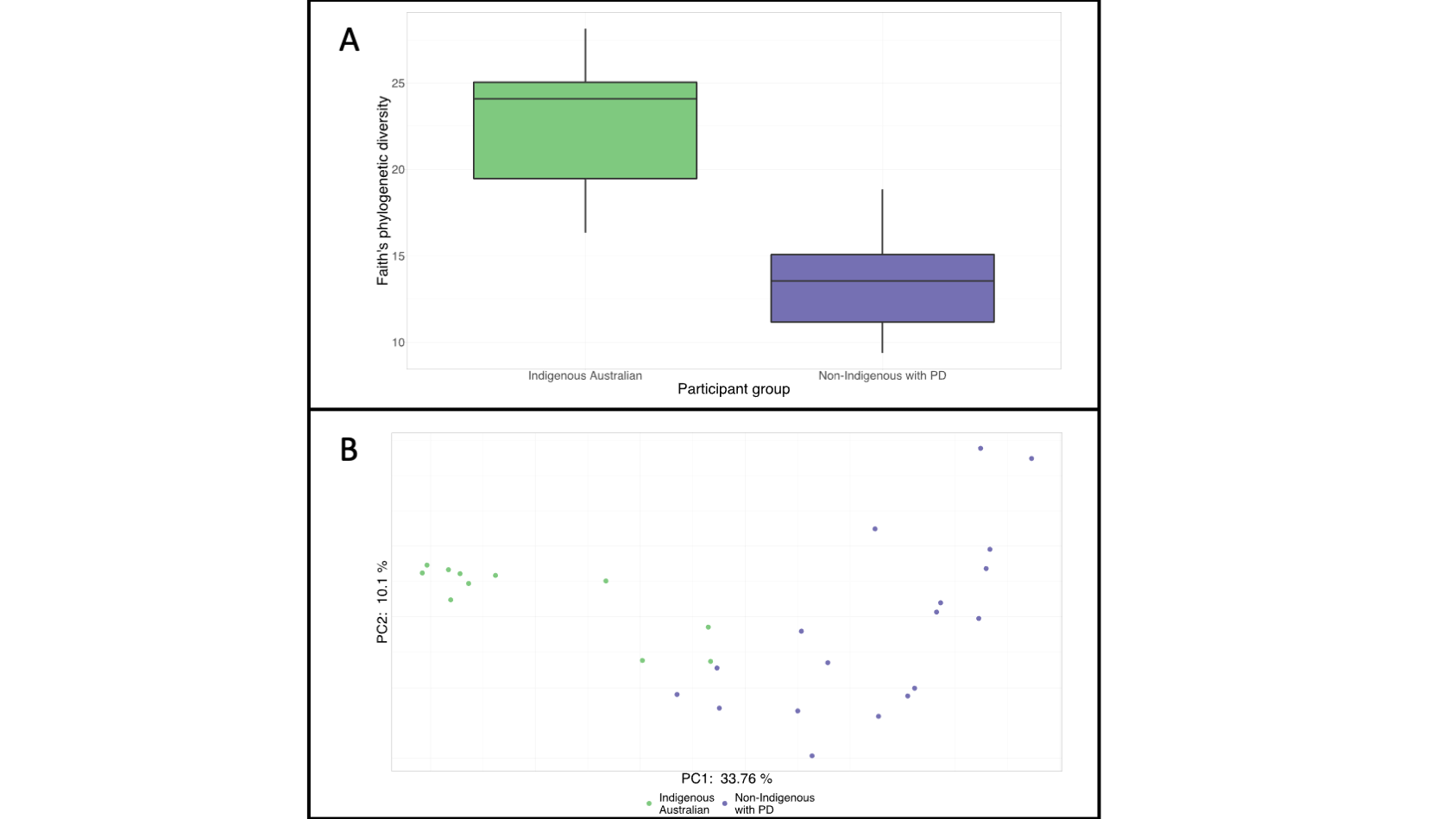


**Figure S3. Significant differences in dental calculus microbiota and composition between Indigenous and non-Indigenous individuals with PD.** (A) Faith’s phylogenetic diversity subsampled to 10,000 sequences per sample. Samples from Indigenous Australians (IPD) have significantly higher diversity than samples from non-Indigenous individuals with PD (NPD) (Kruskal-Wallis H=17.1, p=3.5 x 10^-5^). (B) Principal coordinates analysis (PCoA) of unweighted UniFrac distances, subsampled to 10,000 sequences per sample. Samples from IPD (green) cluster towards one end of PC1 and differ significantly in composition from samples from NPD (purple) (PERMANOVA pseudo-F=9.26, p=0.001).


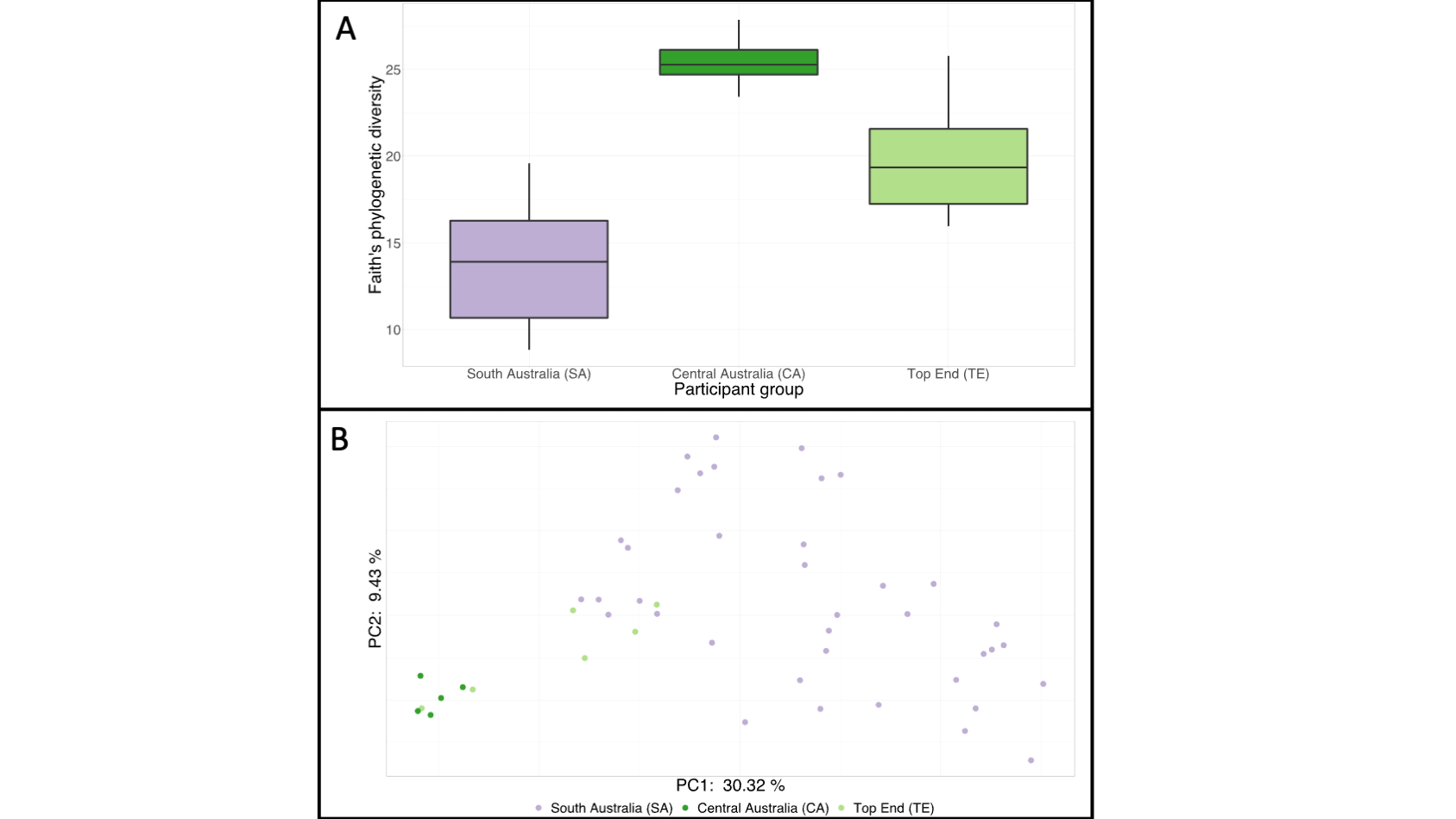


**Figure S4. Differences in dental calculus microbiota according to location.** (A) Faith’s phylogenetic diversity subsampled to 10,000 sequences per sample, showing highest alpha diversity in samples from Central Australia, followed by the Top End and then South Australia (non-Indigenous participants). (B) Principal coordinates analysis (PCoA) of unweighted UniFrac distances, subsampled to 10,000 sequences per sample, showing clustering of Central Australian samples together. Nevertheless, we interpret these results with caution due to the small number of samples available from the Top End (n=6) and Central Australia (n=5) locations.

**Table S5. List of features uniquely found in Indigenous Australians living in the Top End region of Australia**

Refer to Table S5 Excel spreadsheet (TableS5_TopEnd_Unique.xlsx).

**Table S6. List of features uniquely found in Indigenous Australians living in Central Australia**

Refer to Table S6 Excel spreadsheet (TableS6_CentralAus_Unique.xlsx).

References

Anderson, M.J. (2001). A new method for non-parametric multivariate analysis of variance. Austral Ecol. *26*, 32–46.

Benjamini, Y., and Hochberg, Y. (1995). Controlling the False Discovery Rate: A Practical and Powerful Approach to Multiple Testing. J. R. Stat. Soc. Ser. B Methodol. *57*, 289–300.

Bolyen, E., Rideout, J.R., Dillon, M.R., Bokulich, N.A., Abnet, C.C., Al-Ghalith, G.A., Alexander, H., Alm, E.J., Arumugam, M., Asnicar, F., et al. (2019). Reproducible, interactive, scalable and extensible microbiome data science using QIIME 2. Nat. Biotechnol. *37*, 852–857.

Caporaso, J.G., Lauber, C.L., Walters, W.A., Berg-Lyons, D., Huntley, J., Fierer, N., Owens, S.M., Betley, J., Fraser, L., Bauer, M., et al. (2012). Ultra-high-throughput microbial community analysis on the Illumina HiSeq and MiSeq platforms. ISME J. *6*, 1621–1624.

Eisenhofer, R., Minich, J.J., Marotz, C., Cooper, A., Knight, R., and Weyrich, L.S. (2019). Contamination in Low Microbial Biomass Microbiome Studies: Issues and Recommendations. Trends Microbiol. *27*, 105–117.

Escapa, I.F., Chen, T., Huang, Y., Gajare, P., Dewhirst, F.E., and Lemon, K.P. (2018). New Insights into Human Nostril Microbiome from the Expanded Human Oral Microbiome Database (eHOMD): a Resource for the Microbiome of the Human Aerodigestive Tract. MSystems *3*.

Faith, D.P., and Baker, A.M. (2007). Phylogenetic diversity (PD) and biodiversity conservation: some bioinformatics challenges. Evol. Bioinforma. Online *2*, 121–128.

Janssen, S., McDonald, D., Gonzalez, A., Navas-Molina, J.A., Jiang, L., Xu, Z.Z., Winker, K., Kado, D.M., Orwoll, E., Manary, M., et al. (2018). Phylogenetic Placement of Exact Amplicon Sequences Improves Associations with Clinical Information. MSystems *3*.

Kruskal, W.H., and Wallis, W.A. (1952). Use of Ranks in One-Criterion Variance Analysis. J. Am. Stat. Assoc. *47*, 583–621.

Lozupone, C., and Knight, R. (2005). UniFrac: a New Phylogenetic Method for Comparing Microbial Communities. Appl. Environ. Microbiol. *71*, 8228–8235.

Mandal, S., Van Treuren, W., White, R.A., Eggesbø, M., Knight, R., and Peddada, S.D. (2015). Analysis of composition of microbiomes: a novel method for studying microbial composition. Microb. Ecol. Health Dis. *26*.

Salter, S.J., Cox, M.J., Turek, E.M., Calus, S.T., Cookson, W.O., Moffatt, M.F., Turner, P., Parkhill, J., Loman, N.J., and Walker, A.W. (2014). Reagent and laboratory contamination can critically impact sequence-based microbiome analyses. BMC Biol. *12*.

Weyrich, L.S., Farrer, A.G., Eisenhofer, R., Arriola, L.A., Young, J., Selway, C.A., Handsley‐Davis, M., Adler, C.J., Breen, J., and Cooper, A. (2019). Laboratory contamination over time during low-biomass sample analysis. Mol. Ecol. Resour. *19*, 982–996.
